# Functional analysis of embolism induced by air injection in *Acer rubrum* and *Salix nigra*

**DOI:** 10.3389/fpls.2013.00368

**Published:** 2013-09-24

**Authors:** Peter J. Melcher, Maciej A. Zwieniecki

**Affiliations:** ^1^Department of Biology, Ithaca CollegeIthaca, NY, USA; ^2^Department of Plant Sciences, University of CaliforniaDavis, Davis, CA, USA

**Keywords:** embolism refilling, air-pressurization, hydraulic conductivity, Granier sap flow probes, xylem

## Abstract

The goal of this study was to assess the effect of induced embolism with air injection treatments on the function of xylem in *Acer rubrum *L. and* Salix nigra* Marsh. Measurements made on mature trees of *A. rubrum* showed that pneumatic pressurization treatments that created a pressure gradient of 5.5 MPa across pit membranes (Δ*P*_pit_) had no effect on stomatal conductance or on branch-level sap flow. The same air injection treatments made on 3-year-old potted *A. rubrum* plants also had no effect on whole plant transpiration. A separate study made on mature *A. rubrum* trees showed that 3.0 and 5.5 MPa of Δ*P*_pit_ values resulted in an immediate 100% loss in hydraulic conductance (PLC) in petioles. However, the observed change in PLC was short lived, and significant hydraulic recovery occurred within 5–10 min post air-pressurization treatments. Similar experiments conducted on *S. nigra* plants exposed to Δ*P*_pit_ of 3 MPa resulted in a rapid decline in whole plant transpiration followed by leaf wilting and eventual plant death, showing that this species lacks the ability to recover from induced embolism. A survey that measured the effect of air-pressurization treatments on seven other species showed that some species are very sensitive to induction of embolism resulting in leaf wilting and branch death while others show minimal to no effect despite that in each case, the applied Δ*P*_pit_ of 5.5 MPa significantly exceeded any native stress that these plants would experience naturally.

## INTRODUCTION

Tension driven sap transport in plants is powered by evapotranspiration (Dixon and Joly, 1894). This requires that the sap within xylem conduits remain continuous in order to use the cohesive properties of water that allow upward transport against the opposing forces of gravity and hydraulic resistance (Pickard, 1981). Although spontaneous cavitation of water under tension is highly improbable under conditions existing in the xylem (Pickard, 1981), it is common that xylem transport capacity is reduced from embolisms that form either from menisci failure at air water interfaces of bordered pit membranes, or from preexisting air pockets located within xylem conduits (Tyree and Sperry, 1989; Sperry et al., 1996). Menisci failure is thought to depend on the severity of water stress experienced by a plant and from morphological properties of the xylem (Choat et al., 2008). Large variation in the ability to withstand negative pressures exists in plants and it has been shown that some species evolved to tolerate relatively large tensions before embolism starts to affect water transport (-3.5 MPa in some halophyte species like *Rhizophora mangle* L.; Scholander et al., 1964; Sperry et al., 1988b; Melcher et al., 2001) and even down to about –6.7 MPa for a desert shrub *Heteromeles arbutifolia* (Lindl.) M. Roem (Jarbeau et al., 1995). However, for many temperate North American tree species, sap pressures ranging from -3 to -4 MPa would significantly impair the water transport system via massive embolism formation. Recently, it was demonstrated that embolism, measured using acoustic techniques, was the main cause for reduced leaf hydraulic conductance (Johnson et al., 2012). It follows that increased embolisms result in increased leaf-level water stress causing a drop in leaf water potential, reduced stomatal conductance, decreased net photosynthetic gain, leaf wilting, and even leaf death, thus understanding plant responses to hydraulic failure is of great importance.

Embolism formation is more or less stochastic in that it is difficult, if not impossible, to pinpoint exactly where an embolism will form within a stem prior to its occurrence. This imposes limits to the types of studies that can be conducted and thus many reports rely on methods that artificially induce embolism in plants. Based on the assumption that aspiration of air through bordered pit membranes (air seeding) is a major cause of embolism formation and its spread across and along the stem (Choat et al., 2008), an embolism induction method was developed using a pneumatic stem pressurization system (Sperry and Tyree, 1988). Although this method has been used frequently to construct vulnerability curves, it is interesting to ask if it is also feasible to use this technique to study changes in the physiological responses of plants subjected to artificially induced embolism compared to natural water stress induced embolism, especially in the new view of plants being able to refill vessels after they are embolized.

Embolism formation was thought to be irreversible (Tyree and Sperry, 1988). More recent studies have demonstrated that some plant species possess mechanisms to heal embolized vessels even during times when water potentials of plant tissues remain negative. The ability of plants to remove embolisms under tension changes the perception that embolism formation in the xylem of plants is permanent. However, embolism removal, or refilling, when xylem water potentials (Ψ_xp_) are negative (Borghetti et al., 1991; Salleo et al., 1996; Canny, 1997; McCully, 1999; Tyree et al., 1999; Melcher et al., 2001; Clearwater and Clark, 2003; Brodersen et al., 2010; Nardini et al., 2011; Zwieniecki et al., 2013) is not ubiquitous in plants. For example, *Acer negundo* L. lacks the capacity to refill embolism under certain water stress conditions (Hacke and Sperry, 2003). Other studies demonstrate that refilling only occurs after significant reduction of water stress and with limited transpiration, as observed using magnetic resonance imaging (MRI) in grapevines (Holbrook et al., 2001). Direct observations of dynamic changes in water content in the xylem using MRI (Holbrook et al., 2001; Clearwater and Clark, 2003; Scheenen et al., 2007; Zwieniecki et al., 2013) and high-resolution computer tomography (Brodersen et al., 2010), provide strong *in vivo* evidence that refilling in plants occurs under moderate water stress conditions. Kaufmann et al. (2009) used MRI on the roots of *Zea mays* seedlings and observed functional refilling occurred in these seedlings only after re-watering and when plants where under dim light conditions (Kaufmann et al., 2009). The question remains if plants can remove embolisms such that functional hydraulic connections are made during times of moderate plant stress and when exposed to natural sunlight conditions.

The focus of this report is on studying changes in water transport capacity in plants subjected to induced embolism. The level of induced embolism from pneumatic pressurization treatments is independent from levels of plant water stress conditions, thus the instantaneous measure of the level of embolisms within a sample may exceed that of the predicted embolism level from vulnerability curves for a given tension. In theory, air-pressurization methods should allow one to study the biological responses of plants when exposed to embolism. Here we describe the use of air-pressurization treatments to induce embolism and report on the dynamic effects on water transport, stomatal conductance, and the percent loss of hydraulic conductivity (PLC) to determine the feasibility of the air injection method in physiological studies. We use three independent methods to estimate water fluxes (1) heat dissipation probes and (2) changes in stomatal conductance (*g*_s_) measured on mature trees of *A. rubrum*, and (3) whole plant transpiration measured on potted plants of *A. rubrum* and *Salix nigra* measured using an analytical balance. We chose to focus this study on *A. rubrum* and *S. nigra* plants because it had been previously demonstrated that *A. rubrum *had the capacity to refill embolisms (Zwieniecki et al., 2013) and from preliminary tests, *S. nigra* seemed to lack the capacity to tolerate air injection pressures above 3.0 MPa.

## MATERIALS AND METHODS

### PLANT MATERIAL

All measurements were conducted at the Harvard Forest Research Station located in Petersham, MA. Data were collected during the summer months (June to August). Tree ages varied from young adult trees that were about 30 years old (15-m tall trees); younger trees that were 15–20 years old (5-m tall); and 3-year-old potted saplings (1–2 m tall). Scaffolding was used to provide access to the mid-canopy branches of the young adult trees of *A. rubrum* and to conduct various branch-level manipulation studies. All measurements made on *S. nigra* were made on 3-year-old potted saplings. The potted saplings for both *A. rubrum* and *S. nigra* were obtained from Lawyer Nursery (Plains, MT, USA) in early spring. Trees were planted into 7.6-l pots filled with commercial grade potting soil and were grown in the field and exposed to natural light, rainfall, and temperature conditions. All measurements made on potted plants were made in July to August. If necessary, the potted plants were irrigated to maintain predawn water potentials above -0.5 MPa.

### DIURNAL *g*_s_, *E*, Ψ_xp_ AND PLC

Diurnal measurements of stomatal conductance (*g*_s_), transpiration (*E*), covered leaf water potential (Ψ_xp_), and the percent loss of hydraulic conductance (PLC) in petioles were made on mature *A. rubrum* trees. Stomatal conductance and *E* were obtained using a steady state promoter (LI-COR1600, Lincoln, NE, USA). At about 2-h intervals, five mature leaves were sampled from five different trees (25 leaves). Stem water potentials (Ψ_xp_) were estimated by measuring the balancing pressure of a separate subset of leaves (*n* = 5) using a pressure chamber system (PMS Instrument Co. Corvallis, OR). These leaves were covered with plastic bags and aluminum foil at dawn to ensure that the leaf water potentials were in close equilibration with the stem water potential (Begg and Turner, 1970; Turner and Long, 1980; Melcher et al., 1998).

Diurnal PLC was determined on petioles collected from fully expanded mature leaves located mid-canopy on south facing branches from five mature *A. rubrum* trees. At ~2 h intervals, five petioles (about 5-cm long) were excised under water in the field with razor clippers and transported to the laboratory in a container filled with tap water (the laboratory was about 100 m away from the trees). In the lab, 1-cm long petiole segments were re-cut under water from each petiole using fresh razor blades and attached to a hydraulic apparatus (Sperry et al., 1988a). Care was taken during petiole attachment to the hydraulic apparatus to ensure that embolisms were not inadvertently pushed out of open conduits since the petiole segments were shorter then measured conduit lengths. The flow rate (*J*_v_) of perfusion solution through the petiole was measured using an analytical balance (±0.01 mg, Sartorius Model #R160P, Gottingen, Germany) interfaced to a computer. A 10 mM KCl perfusion solution was used in all hydraulic experiments to reduce potential fluctuations that can result from bordered pit membrane ionic responses (Zwieniecki et al., 2001). Petioles were supplied a constant pressure head (Δ*P*) of 1.5 kPa and the native petiole specific conductivity (*k*_n_) was calculated as:

(1) $$k_{n}=J_{v}/\Delta P\times L,$$

where *L* is the petiole length. After determination of *k*_n_, a 200-kPa pressure flush was applied to each petiole segment using degassed 10 mM KCl solution for 2 min and repeated three times to restore *k*_n_ to its maximum hydraulic conductivity value (*k*_m_). After flushing, petioles were reattached to the hydraulic apparatus to measure *k*_m_ under the same hydraulic conditions used to determine *k*_n_. The PLC of the petiole (*k*_PLC_) was calculated as *k*_PLC_ = 100 × (1 - *k*_n_/*k*_m_).

### AIR-PRESSURIZATION – STEM PLC

The effects of air pressurization on PLC were measured on 3-year-old stems of *A. rubrum* plants collected from the field. Large leafy branches (1.5 m long) were excised from trees early in the morning (around 6:00 am). They were placed into plastic bags that contained moist paper towels and taken to the lab. Shorter, 0.15-m long unbranched stem segments were then re-cut from the large branches under tap water and used for air-pressurization experiments. Initial stem hydraulic conductivity (*k*_h_) was determined (as described for petioles). Then nitrogen gas was forced into the distal end of each stem segment for 1 min. This was achieved by attaching the distal end of each stem segment to a high pressure compression fitting that was attached to pressure delivery system. During pressurization, the entire stem and fitting was placed under water to prevent the non-pressurized stem end from drying out. Gas pressure was released rapidly and the stem was allowed to degas in a container filled with clean perfusion solution for 3 h. After degassing, it was necessary to apply 10–20 kPa of hydraulic suction for 8 min to remove gas from the gas pressurized end. We found this to be necessary because it removed a 10–20% loss in stem *k*_h_ that was observed to occur at relatively low applied gas pressures (<0.5 MPa) compared to when hydraulic suction was not applied to the gas pressurized end of the stems. Following suction, stems were reattached to the hydraulic apparatus to measure *k*_h_ post gas pressurization and PLC was determined as described for petioles. The hydraulic protocol used for the stems differed slightly from that used for the petioles, in that a hydraulic head of 10 kPa was used to measure *k*_h_ before and after each air-pressurization treatment. Also, a series of four 10-min, 400 kPa hydraulic pressure flushes, were used to achieve maximum stem hydraulic conductivity. This procedure was repeated at each pressure step until *k*_h_ was reduced by 100%.

### AIR-PRESSURIZATION – EFFECT ON SAP FLOW USING GRANIER STYLE PROBES

Measurements of sap flow using heat dissipation type “Granier” probes were made on small diameter branches (about 5 cm). This was achieved by the authors by constructing probes that had both the reference and the heated components installed on a single in-line probe, with reference and heated parts located on opposite ends of each 25 cm long probe. This allowed measurements to be made in a single insertion point into a branch. Instead of making axial drill holes, as in traditional two-probe systems, only one longitudinal hole was made through a branch junction into the pith using a 30 cm long drill bit. The probe was inserted into this hole such that it was in line with the main portion of the stem. To ensure good thermal contact between the probe and the xylem conducting tissue, each probe was covered with a thin layer of high conductive thermal paste before inserting the probes into the stems. Constant voltage was supplied to the upstream ends of the probes to maintain a constant temperature. Temperature was measured on both ends using fine wire copper-constantan thermocouples interfaced to a multiplexer and a Campbell Scientific datalogging system (CR10X, Logan, UT, USA). The temperature difference between the upstream and downstream ends was used to calculate sap flow rates through the branches.

**FIGURE 1:**
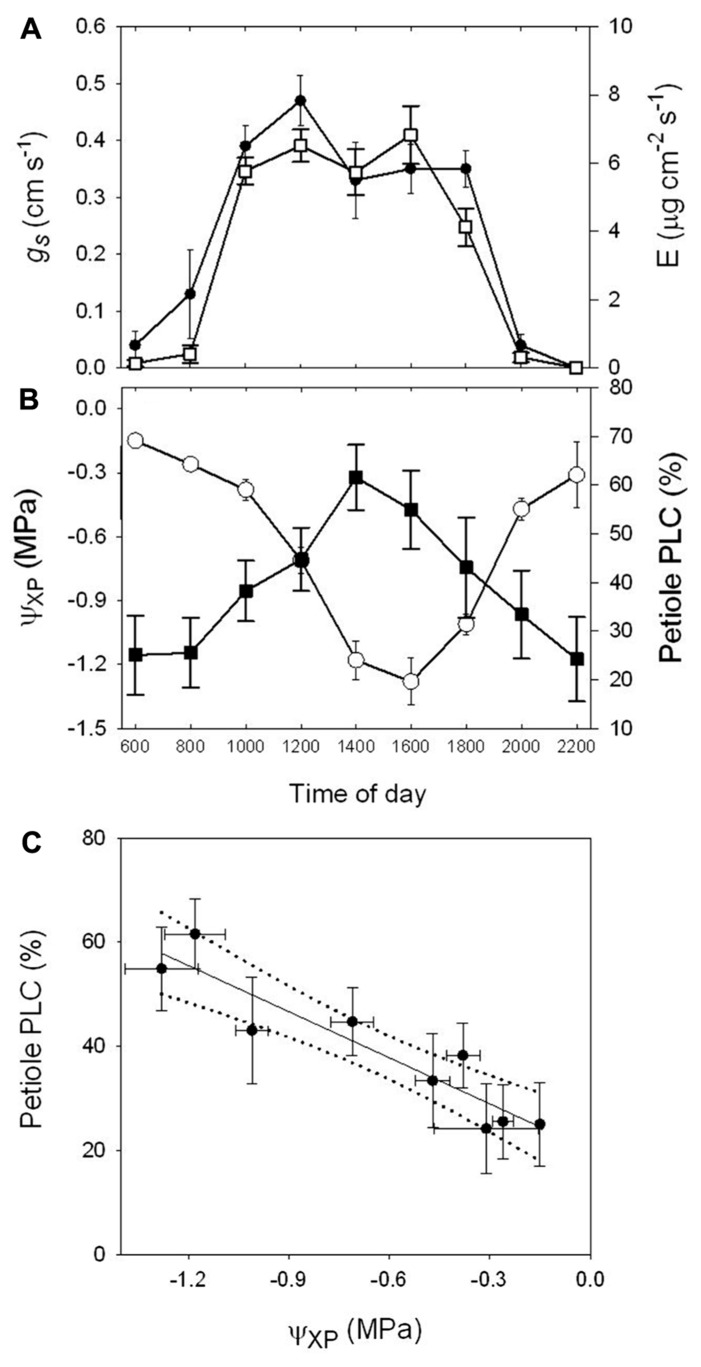
**(A)** Diurnal trends in stomatal conductance (*g*_s_, *n* = 15) and transpiration rate (*E*, *n* = 15), **(B)** covered leaf water potential (Ψ_xp_, *n* = 10) and the percent loss in hydraulic conductance of petioles (PLC, *n* = 10) of *A. rubrum* measured on a sunny day from mature trees. **(C)** Regression analysis of PLC_pet_ to Ψ_xp_ (*y* = -29.47*x* + 20.14, *R*^2^ = 0.87) of data from **(B)**. Each data point represents the pooled average values measured from three mature trees located near each other.

Simultaneous sap flow measurements were made using six probes, installed into six south facing branches located mid canopy on three mature trees. A scaffolding system provided access to the branches. Data were recorded every second and averages every 10 min. Three branches were used as positive controls and three branches were pressurized. Before pressurizing each stem with nitrogen gas, covered leaf water potentials (Ψ_xp_) were measured using a Scholander pressure bomb on three leaves that were covered in plastic bags and aluminum foil at dawn. The average value of Ψ_xp_ was used to determine how much gas pressure to apply in order to create a 5.5 MPa pressure gradient across the xylem pit membranes (Δ*P*_pi__t_) [Δ*P*_pit_ = *P*_gas_ - Ψ_xp_]. Stems were pressurized at about 10:00 am for 2 min using a pressure delivery system attached to an excised side branch located about 1–1.5 m upstream of the sap flow probes. We chose 10:00 h for the application of pneumatic treatments a bit arbitrarily, but mainly because we knew that the plants were under negative water potentials at this time and that PLC levels, due to natural embolism, was high but not at maximum values as determined from diurnal PLC studies on *A. rubrum* (**Figure 1B**). We chose to pressurize the stems for 2 min because we wanted to ensure that the gas penetrated all the vessels near the site of air injection. It took about 1 min to hear the gas escape from downstream petioles as indicated by snapping, crackling, and popping sounds. Sap flow was measured continuously for several days before and after air pressurization. It should be noted that during gas pressurization, the applied gas infiltrated the branch and traveled several meters from the insertion point to the petioles and leaves. This was known because gas would cause snapping or crackling sounds from petioles during pressurization.

### AIR-PRESSURIZATION – EFFECT ON GS AND Ψ_L_

The effect of air-pressurization on stomatal conductance was determined by inducing Δ*P*_pit_ values of 5.5 MPa into excised side branches about 1.5–2.0 m upstream of the measured leaves. Stomatal conductance (*g*_s_) and leaf transpiration (*E*) were measured using a steady state porometer (LI-COR1600, Lincoln, NE, USA). Simultaneous values of Ψ_xp_ were obtained using a pressure chamber system on covered leaves. We compared three branches that were air-injected to three branches that were left intact “control branches.” We also made measurements on two excised branches that were left in the shade of the tree as our “negative control.” Measurements on the three treatments were made together to provide comparative analysis because *g*_s_, *E*, and Ψ_xp_ change diurnally.

### AIR-PRESSURIZATION – EFFECT ON WHOLE PLANT TRANSPIRATION

Three-year-old *A. rubrum* and *S. nigra* potted saplings were used for measuring the effects of air-pressurization on whole plant transpiration. Pressure collars were placed around the main stem of each plant before the leaves appeared in the spring to allow for air-pressurization treatments during the summer. After several months of summer growth plants were transported from the field to a greenhouse and placed on a high capacity analytical balance (Sartorius ±0.1 g) that was interfaced to a computer. The pots were double wrapped in plastic bags and the plastic was taped around the main stem in an effort to minimize evaporation from the pot. The change in weight was recorded every second and the average was collected every minute. Before air-pressurization, each plant was removed from the balance and several axial slits were made through the xylem using a razor blade to allow for the gas to penetrate the water conducting tissue (Sperry et al., 1988a). If the plant had a pressure collar already attached to it, then it was used to apply pressurization of gas, otherwise we attached a self-built split-chamber pressure collar to the main stem. Before pressurizing each stem, covered leaf water potential (Ψ_xp_) was measured on three leaves using a Scholander pressure bomb. The average value of Ψ_xp_ was used to determine Δ*P*_pit_. Each plant was pressurized for 2 min. After the pressurization treatment, each plant was returned to the analytical balance and their weights were continuously monitored until the next morning and in some cases for several days. All plants were pressurized in the morning hours (9:30–11:00 am). This procedure was repeated on a total of five plants of *A. rubrum* and three plants for *S. nigra*. Additional experiments were also performed on *S. nigra* plants that were subjected to air-pressurization pressures to generate Δ*P*_pit_ values of 0.5, 1.0, 2.0, 3.0 MPa that were administered to different plants to determine the pressure required to stop transpiration and determine if plants could refill embolisms after pressurization treatments. Control plants were not pressurized but they also had slits made into their xylem using a razor blade.

### AIR-PRESSURIZATION – PETIOLE RECOVERY

Changes in specific petiole conductivity (*k*_pet_) were measured before and after air-pressurization treatments of 15 young *A. rubrum* trees at 10 am. Five trees were used for each Δ*P*_pit_ treatment, 0 (control), 3.0, and 5.5 MPa. Air-pressurization followed the same procedure used for the sap flow probe studies. Petioles were excised underwater using razor clippers and transported back to the laboratory while remaining in water. In the lab *k*_pet_ was measured using a hydraulic apparatus following the same procedure used to determine diurnal patterns of PLC.

### AIR-PRESSURIZATION – PLANT SPECIES SURVEY

Eight species, *Parthenocissus tricuspidata* (Siebold & Zucc.) Planch (vine), *Sambucus nigra* L. (shrub)^1^, *Cornus rugosa* Lam. (shrub), *Magnolia* spp. L. (tree), *Fraxinus americana* L. (tree), *Quercus rubra* L. (tree), *Ulmus americana* L. (tree), and *A. rubrum* (tree), were used to investigate how air-pressurization treatment affect stomatal conductance. Five branches, each from a separate plant, were either pressurized with gas for 2 min, not pressurized (positive controls) and excised from the branch and not pressurized (negative control). For pressurization treatments, gas pressurization was achieved by excising a side branch and attaching this with a compression collar that was attached to a pressure delivery system. Gas delivery pressure was increased at a rate of about 0.5 MPa min^-^^1^ and when the desired air injection pressure of 5.0 MPa was achieved, the pressure was maintained for 2 min. Stomatal conductance was measured using a steady state porometer (LI-COR1600, Lincoln, NE, USA), five times every 2 min for a 10-min period prior to pressurization treatments. Five stomatal conductance measurements were also measured the following day at the same time to determine if the pressurization treatment impacted stomatal function.

## RESULTS

The diurnal transpiration rate (*E*), stomatal conductance (*g*_s_), covered leaf water potential (Ψ_xp_), and the percent loss in petiole hydraulic conductivity (PLC) were measured for a 24-h period. Measurements were collected from mid-canopy leaves on young adult trees about 20-m tall on a sunny day (**Figures 1A,B**). Peak *E* and *g*_s_ occurred between the hours of 10:00 am and 4:00 pm with corresponding Ψ_xp_ of -0.5 to -1.0 MPa with respective native PLC values of 45% and 55%. The field trees showed a native permanent level of embolism (predawn values) of about 25%. We also found a strong correlation between petiole PLC and Ψ_xp_ from regression analysis (*y* = -29.47*x* + 20.14; *r*^2^ = 0.87; **Figure 1C**).

**FIGURE 2:**
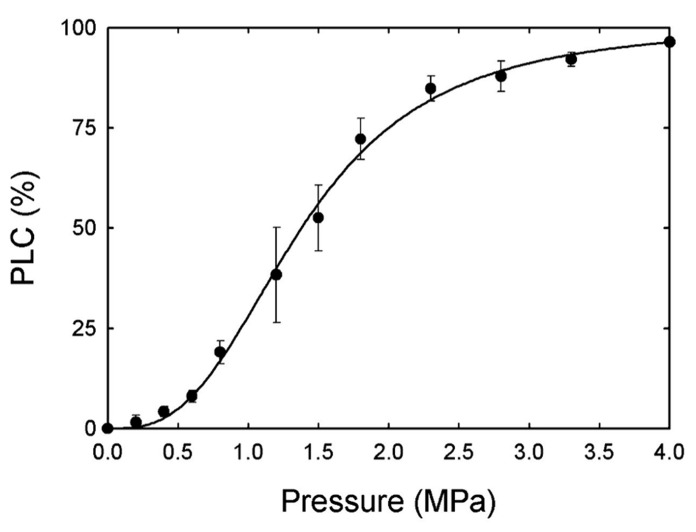
**Average percent loss in hydraulic conductance (PLC) was measured on current year stems of *A. rubrum* after applying pneumatic pressure (PLC_50_ = 1.4 MPa).** The minimum pressure required to reduce hydraulic conductivity by 95% was 4.0 MPa (*n* = 6).

The average percent loss in hydraulic conductance (PLC) was measured on current year stems of *A. rubrum* after applying pneumatic pressure (PLC_50_ = 1.4 MPa). The minimum pressure required to reduce hydraulic conductivity by 95% was 4.0 MPa (**Figure 2**). Due to the fact that *S. nigra* has very long vessels we were unable to measure vulnerability curves for this species or to determine how PLC changes diurnally due to potential errors that can arise from hydraulic measurements made on plants with long vessels (Wheeler et al., 2013).

**FIGURE 3:**
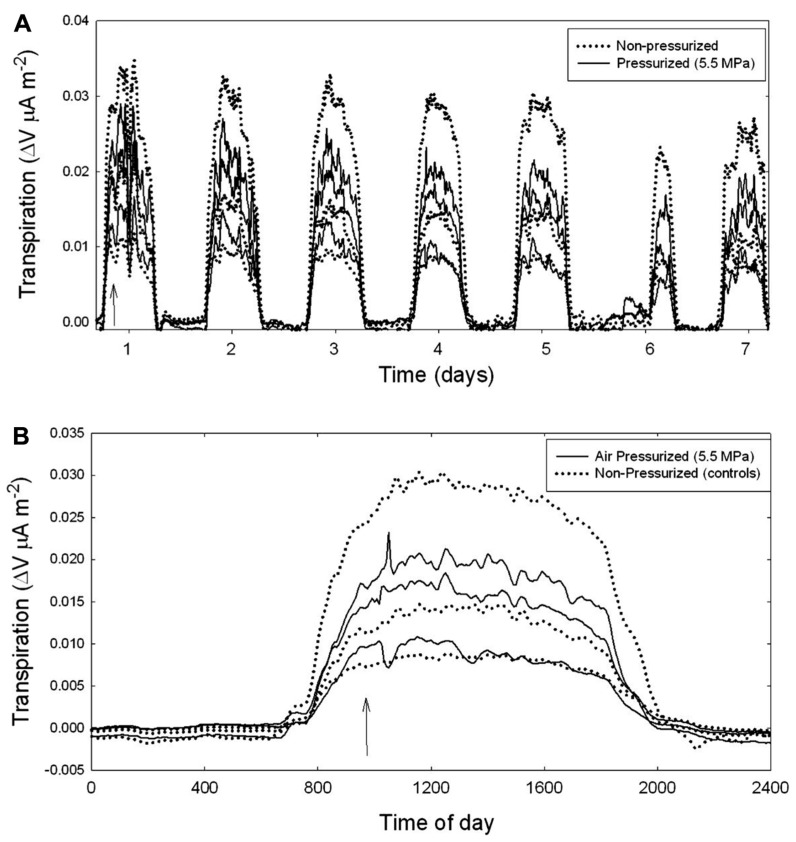
**Branch-level sap flux measured on mature *A. rubrum* trees using heat dissipation sap flow probes.** **(A)** Several consecutive days of measurements are shown after applying 5.5 MPa of Δ*P*_pit_ (solid lines) compared to non-pressurized control branches (dotted lines). The arrow indicates when gas pressure was applied to the xylem. Note that on day 6 it was raining in the morning resulting in low flow rates for all branches measured. **(B)** The effects of air-pressurization on sap flow shown for a 24-h period. All branch-level measurements are standardized by total leaf area.

The flow of sap through branches attached to mature trees was measured using Granier style sap flow probes (**Figure 3**). Data were collected over many days after each pressurization treatment (**Figure 3A**). Sap flow rates are shown for both the non-pressurized, control branches (dotted lines), and for branches that were pressurized to Δ*P*_pit_ values of 5.5 MPa (solid line). Pressurization of all treated branches was done at about 10 am in the morning (**Figure 3A**). An arrow indicates when air-pressurization occurred (**Figure 3B**). Note that in **Figure 3A**, it was raining in the morning on day 6 (Julian day 226) resulting in reduced sap flow for all branches. **Figure 3B** shows the effect of injecting nitrogen gas on branches (solid line) compared to controls (dotted lines) for a 24-h period. There were no measurable effects on the flow of sap from air injection treatments that induced Δ*P*_pit_ values of 5.5 MPa. After about 30 s of applied pressures many petioles would produce a popping sound and gas would escape out of them as indicated by the production of small bubbles of a white substance.

**FIGURE 4:**
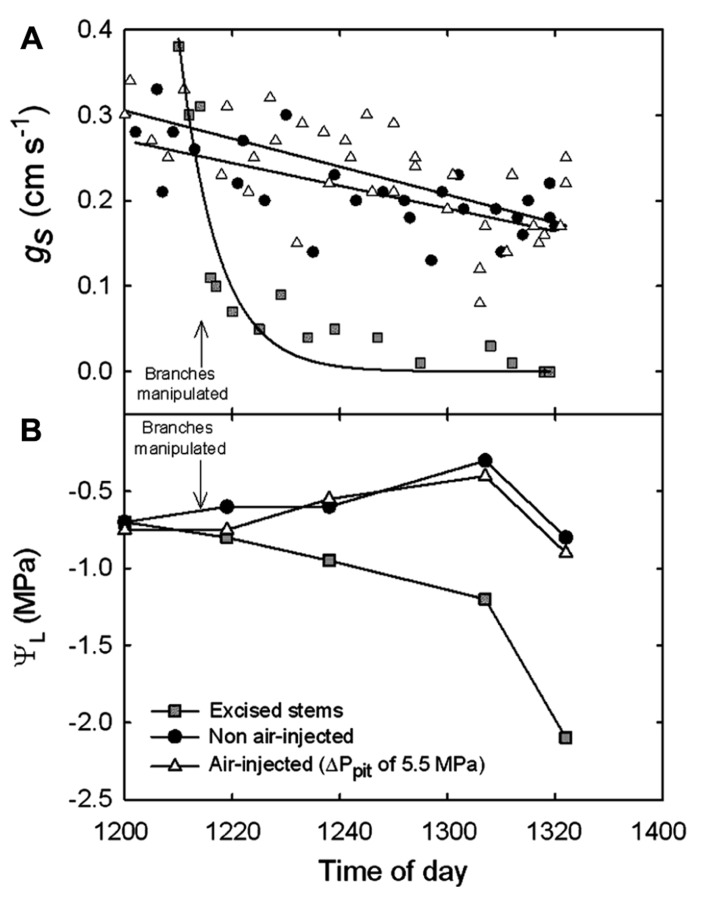
**(A)** Stomatal conductance (*g*_s_) measured on *A. rubrum* leaves attached to stems before and after pneumatic pressurization treatment with Δ*P*_pit_ of 5.5 MPa (triangles) compared to non-injected control branches (circles) and from excised branches (squares). **(B)** Xylem pressure potentials (Ψ_xp_) measured from leaves covered in plastic bags that were attached to controls and manipulated branches during the experiment.

Leaf level measurements of *g*_s_, and uncovered leaf water potentials (Ψ_L_) were measured before and after applying Δ*P*_pit_ values of 5.5 MPa into branches attached to mature trees of *A. rubrum* (**Figure 4**). Measurements of *g*_s_ were made on leaves attached to control (circles), air-pressurized (triangles), and branches that were completely excised from the tree and left in the shade (squares). Corresponding covered Ψ_L_ are shown (**Figure 4B**). Values of Ψ_L_ decreased compared to the controls in the excised branches only. Each data point represents an *n* = 1 because we did not want to remove too many leaves from the test plants during measurements of *g*_s_.

**FIGURE 5:**
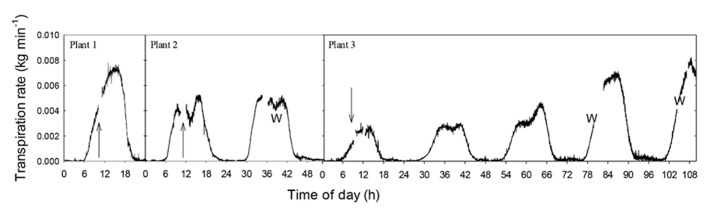
**Whole plant transpiration of potted plants of 2-year-old *A. rubrum* trees.** Data were measured using an analytical balance before and after injection of gas that created Δ*P*_pit_ values of 5.5 MPa in the main stem of the treated plants. The arrows indicate when pneumatic treatment was applied. The *W* represents when the plants were removed from the balance for watering.

**FIGURE 6:**
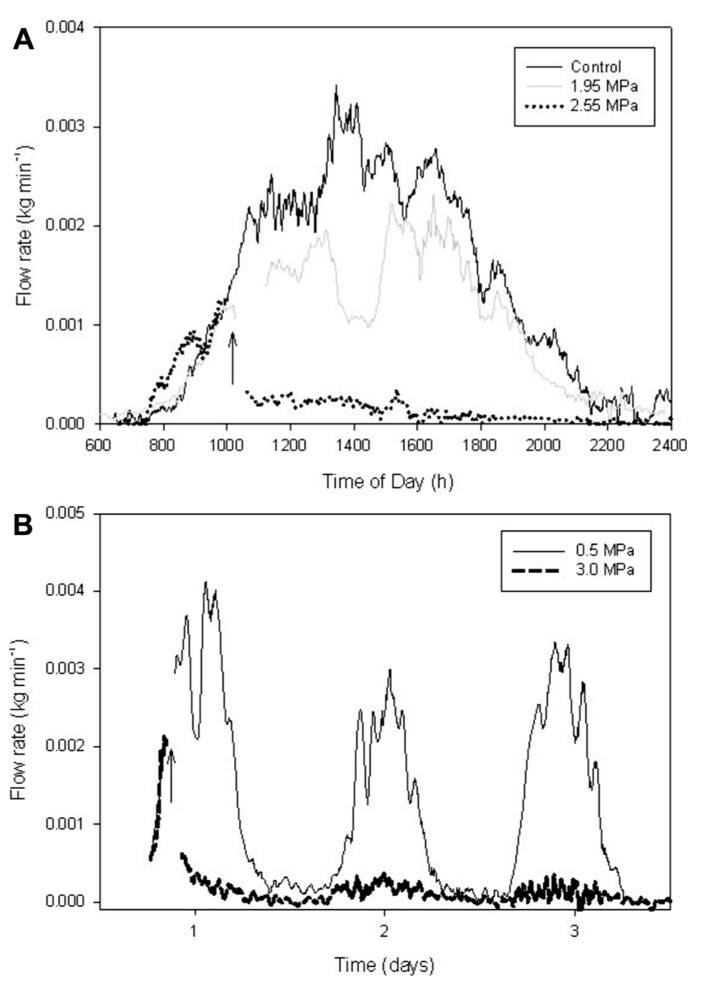
**Whole plant transpiration measurements made on potted plants of 3-year-old *S. nigra* trees collected using an analytical balance before and after air-pressurization treatments.** The arrows indicate when pneumatic treatments were applied. **(A)** Shows three different plants pressurized with three different Δ*P*_pit_ air injection pressure treatments measured over a 24-h period. **(B)** Shows two plants subjected to Δ*P*_pit_ pressures of 0.5 and 3.0 MPa. Note that Δ*P*_pit_ pressures over 2.0 MPa resulted in reduced flow and non-recovery in *S. nigra*.

The effect of air injection treatments on whole-plant transpiration was measured on potted plants of *A. rubrum* (**Figure 5**) and *S. nigra* (**Figure 6**). *A. rubrum* plants showed variable response to the application of stem pressurization that resulted in Δ*P*_pit_ of 5.5 MPa. While plant number one seemed to maintain and even increase transpiration rate after application of pressure, plant two, and three experienced a small reduction in mid-day transpiration which was consistent with mid-day transpiration reduction under moderate water stress conditions. Re-watering restored higher transpiration rates in the case of the plant 3. Subjecting *S. nigra* plants to Δ*P*_pit_ treatments greater than 2.55 MPa, rapidly and permanently stopped the flow of sap through the plants (the plants died a few days after the treatment). Whole plant transpiration was monitored for both species after air-pressurization for several days in order to determine if there were any longer-term effects of air-pressurization on whole plant transpiration. *S. nigra* plants pressurized to Δ*P*_pit_ values of 2.55 MPa, died. All other plants for both *A. rubrum* and *S. nigra* survived the pressurization treatments without any visible damage or drop in expected transpiration rates (**Figures 5** and **6**).

**FIGURE 7:**
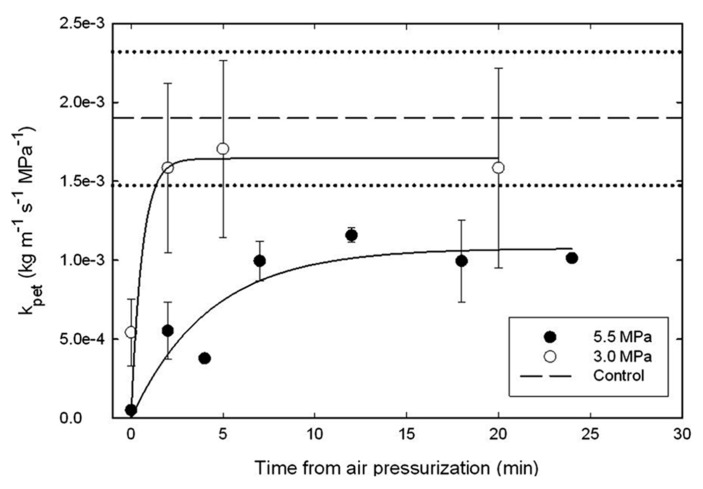
**Changes in petiole specific hydraulic conductivity (*k*_pet_) measured after applying Δ*P*_pit_ treatments of 3.0 (open symbols) and 5.0 MPa (closed symbols) of nitrogen gas through excised side branches located about 1-m upstream of the petioles that were used for *k*_**pet**_ analysis.** Data were compared to non-pressurized controls (dashed lines represent the averages and dotted lines represent the 95% C.I. of the controls). Petioles remained attached to the trees until collected for *k*_pet_ measurements. Note that *k*_pet_ was restored to average control *k*_pet_ values within 2.5 min from the Δ*P*_pit_ 3.0 MPa air-pressurization treatment. It took 5 min to reach about 70% of average *k*_pet_ for control branches for 5.5 MPa of Δ*P*_pit_ pressure treatments. Five trees were used for each measurement treatment, e.g., 15 trees in total.

Petiole *k*_s_ was monitored for 30 min after pressurizing plants to generate a Δ*P*_pit_ value of 5.5 MPa in the xylem of branches of mature *A. rubrum* trees growing in the field (**Figure 7**). The data show that air pressurization reduced *k*_pet_ to values near zero immediately following air-pressurization (time zero). Within 2.5 min, *k*_pet_ was restored on branches subjected to the 3.0 MPa treatments. In the case of the 5.5 MPa pressurization, it took around 10 min to restore *k*_pet_ to 70% of the average *k*_pet_ measured from control branches (dashed line), and these petioles seemed to remain at about 30% reduced *k*_pet_ values for the 30 min of measurement time.

**Figure 8:**
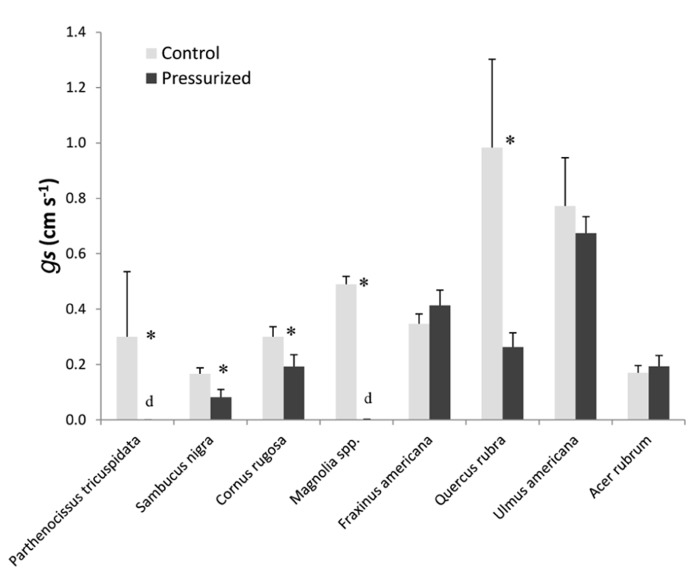
**A survey of plant tolerance to pneumatic pressurization treatments.** All plants were pressurized with 5.5 MPa of gas pressure for 2 min. Repeated measures of stomatal conductance were measured for 10 min before air-pressurization treatments and then the following day. All measurements were made at the same time of the day before and after air-pressurization. Asterisks represent leaves that were wilted the following day. Both days were similar in abiotic conditions (e.g., both days were sunny). For all species, stomatal conductance was also measured on leaves from branches before and 24 h after being excised from the parent tree. Leaves from all excised branches had zero-stomatal conductance (data not shown).

We surveyed various plants to determine the effect of air-pressurization treatments on stomatal conductance (**Figure 8**). We found that applying gas pressures of 5.0 MPa resulted in a total loss of stomatal conductance in *P. tricuspidata* and *Magnolia* spp. and resulted in branch death several days after air-pressurization treatments. Species that only partially recovered (stomatal conductance reduced by 50–80%) were *Sambucus nigra*, *C. rugosa*, and *Q. rubra*. *F. americana*, *U. americana*, and *A. rubrum* showed no effect of air pressurization on stomatal conductance. We also measured changes in stomatal conductance on excised branches for all species surveyed and found that stomatal conductance was zero in all species.

## DISCUSSION

Here we present results from short (minutes) and long-term studies (days) that measure the response of *A. rubrum* and *S. nigra* to air-pressurization treatments. The choice of species was dictated by our expectation that the habit of the species (*A. rubrum* is a tree and *S. nigra* is a large multi-stem shrub) may be a result of their ability to tolerate embolism. In this study all air-pressurization treatments combined the value of applied gas pressure and the measured sap tension required to produce a known pressure gradient across the air water interface at bordered pit membranes (Δ*P*_pit_). We found that Δ*P*_pit_ values of 5.5 MPa had minimal effect on transpiration, stomatal conductance, and sap flow on *A. rubrum* trees. Whereas, Δ*P*_pit_ values greater than 2.5 MPa had devastating effects on *S. nigra* plants with concomitant inhibition of whole plant transpiration and no signs of recovery that eventually resulted in plant death. The difference in response to the air injection treatments of these two species may explain “shrub strategy” that embolism formation results in the loss of the stem function and the necessity to replace it with a new stem. The “tree strategy” of *A. rubrum* might rely on the ability of the plant to not only protect itself from embolism formation but in the event that embolisms occur, it has the ability to restore hydraulic function and avoid catastrophic discontinuity of the xylem sap. However, this simplified view is complicated by the fact that ring-porous species (*Q. rubra*) still showed significant loss of stomatal conductivity 24 h after induction of embolism.

In this study, diurnal embolism levels tracked diurnal leaf-level water potentials in *A. rubrum* (**Figure 1**) as shown previously to occur in *R. mangle* (Melcher et al., 2001). It is easier to understand why more vessels embolize with increasing tension, but if the refilling process is initiated by an embolism event (Secchi and Zwieniecki, 2010), then this implies that refilling might be happening concurrently with increased levels of embolism formation as water potential continues to decrease from morning to mid-day. However, when a plant reaches its minimum diurnal xylem water potential (Ψ_xp_) it is most likely associated with the highest level of cavitation events and the lowest level of refilling capacity. Our data suggest that from 14:00 to 22:00, *A. rubrum* plants were able to recover part of their capacity to transport water. This recovery is associated with an afternoon drop in stomatal conductance, reduced transpiration, and partial recovery of stem water potential. These physiological changes may play an important role in reversing the ratio of cavitation to refilling events and allow plants to recover from embolism starting in the afternoon and continue through the night.

Several physiological mechanisms have been proposed to describe how plants refill embolized conduits when the surrounding tissue is under negative water potentials (Nardini et al., 2011). Holbrook and Zwieniecki (1999) describe that water first enters the conduit lumens followed by refilling of the pit chambers. The geometry of the pit chambers act as pressure valves to ensure that all the small air pockets trapped in the bordered pit chambers refill before simultaneous hydraulic connections are made (Holbrook and Zwieniecki, 1999). Thus, if only the lumens are filled with sap, one cannot assume that hydraulic continuity has been restored with neighboring xylem conduits, or that “functional refilling” has occurred (Kaufmann et al., 2009; Zwieniecki and Holbrook, 2009). Since excising stem segments underwater for assessment of embolism status using hydraulic protocols results in a rapid relaxation in hydraulic tension, this may allow for refilling vessels to establish hydraulic continuity. If these hydraulic connections are made after excision underwater due to tension relaxation, then this would result in false estimates of hydraulic restoration, as seen by Wheeler et al. (2013), and may lead to false estimates of high resistance to embolism (Wheeler et al., 2013). There is a similar issue related to the interpretation of cryo-scanning electron microscope (cryo-SEM; Canny et al., 2001) and X-ray tomography (Brodersen et al., 2010) analysis because they both assess the water status of the vessel lumens and not hydraulic continuity between vessels. It is quite possible that plants continuously refill vessel lumens during times of negative water potentials but that true hydraulic connections only get established when xylem sap tensions are relaxed as previously observed using MRI on roots of *Z. mays* (Kaufmann et al., 2009).

Two approaches were used in this study to address the issue if refilling of vessels results in functional refilling in *A. rubrum* (1) whole plant air-pressurization studies on potted plants with measurements of whole plant transpiration rates and (2) measured changes in fluid flow on stems of mature trees pressurized with gas measured using Granier style sap flow probes. We chose both of these approaches because they do not require destructive sampling techniques and should provide insight on whether the removal of embolisms from pneumatic pressurization treatments results in functional refilling. We induced Δ*P*_pit_ values of 5.5 MPa across bordered pit membranes because this value was shown to reduce hydraulic conductivity by nearly 100% in petioles determined from vulnerability analysis (**Figure 2**). Despite this high level of pressure treatment, we were unable to permanently shut down transpiration in the stems and petioles of *A. rubrum* even for a short period of time following injection of gas. Stomatal conductance and sap flow remained generally unaffected in both the branches of mature trees and on whole plant transpiration measured on potted plants. The dynamics of the restoration of transport function from induced embolism, as determined from PLC measurements, was very fast. The rates are similar, or even faster than the refilling time determined from MRI investigations of natural and pressure induced embolism observed in the same species (Zwieniecki et al., 2013). However, we feel that analysis of embolism refilling from air-pressurization treatments should be taken with caution as determination of PLC requires a prolonged period of “de-gassing” during which time samples remain biologically active and could potentially restore functionality of the hydraulic path from available water. Thus, more studies are needed to better understand the degree that air pressurization alters the native state of the hydraulic path.

The degree that air injection mimics natural formation of embolism in stems, beyond the correlation between PCL curves obtained from branches exposed to tension and those exposed to air-pressurization induced embolism (Sperry et al., 1988a), is still not well understood. Thus it is not clear what happens when gas is forced into the xylem at high pressures and where the water goes when it is displaced during pressurization treatments. In our study, we found that the rate that hydraulic conductance returns to pre air injection levels in *A. rubrum* was very fast (<10 min) thus we were unable to detect any physiological effects from air injection based on stomatal conductance and sap flow measurements that were made at 10 min intervals. However, we did find that the same level of pressures applied to several species resulted in significantly different responses showing that some species are very sensitive to air injection treatments such as the shrub-like species *Sambucus nigra* and *C. rugosa* and the vine *P. tricuspidata*. This study did find support that other tree species such as *F. americana*, and *U. americana* remained fully operational within a short time after pressurization. However, the tree species *Magnolia *spp. and *Q. rubra* did not tolerate air injection very well suggesting that not all tree species possess the capacity to refill induced embolism.

## Conflict of Interest Statement

The authors declare that the research was conducted in the absence of any commercial or financial relationships that could be construed as a potential conflict of interest.

## Footnotes

1 All *S. nigra* refers to *Salix nigra* and all *Sambucus nigra* are not abbreviated throughout the manuscript.
